# Protecting the underscreened women in developed countries: the value of HPV test

**DOI:** 10.1186/1471-2407-14-574

**Published:** 2014-08-08

**Authors:** Raquel Ibáñez, Josefina Autonell, Montserrat Sardà, Nayade Crespo, Pilar Pique, Amparo Pascual, Clara Martí, Montserrat Fibla, Cristina Gutiérrez, Belén Lloveras, Judit Moreno-Crespi, Anna Torrent, Núria Baixeras, María Alejo, Francesc Xavier Bosch, Silvia de Sanjosé

**Affiliations:** Unit of Infections and Cancer; Cancer Epidemiology Research Programme, IDIBELL, Catalan Institute of Oncology (ICO), 08908 L’Hospitalet de Llobregat, Barcelona, Spain; Pathology Department, Consorci Hospitalari de Vic, 08500 Vic, Barcelona, Spain; Sexual and Reproductive Health Centre of Bages-Solsonès, 08240 Manresa Barcelona, Spain; Pathology Department, Hospital General de Granollers, 08402 Granollers Barcelona, Spain; Pathology Department, Hospital Universitari Joan XXIII de Tarragona, 43005 Tarragona, Spain; Clinical Laboratory ICS Tarragona, Molecular Biology Section, Hospital Universitari Joan XXIII de Tarragona. IISPV Rovira i Virgili University, 43005 Tarragona, Spain; Pathology Department, Hospital del Mar, 08003 Barcelona, Spain; Pathology Department, Hospital Universitari Dr. Josep Trueta de Girona. Catalan Institute of Oncology, 17007 Girona, Spain; Sexual and Reproductive Health centre of Mollet del Vallés 08100 Mollet del Vallès, Barcelona, Spain; Pathology Department, Hospital Universitari de Bellvitge, IDIBELL, Catalan Institute of Oncology d’Oncologia 08908 L’Hospitalet de Llobregat, Barcelona, Spain; Pathology Department, Hospital General de L’Hospitalet. 08906 L’Hospitalet de Llobregat, Barcelona, Spain; CIBER Epidemiology and Public Health, Barcelona, Spain

**Keywords:** Human papilloma virus, Cervical cytology, Pap smear, Cervical cancer screening, HC2 testing, HPV test, Sensitivity, Specificity, Underscreened women

## Abstract

**Background:**

Poor attendance to cervical cancer (CC) screening is a major risk factor for CC. Efforts to capture underscreened women are considerable and once women agree to participate, the provision of longitudinal validity of the screening test is of paramount relevance. We evaluate the addition of high risk HPV test (HPV) to cervical cytology as a primary screening test among underscreened women in the longitudinal prediction of intraepithelial lesions grade 2 or worse (CIN2+).

**Methods:**

Women were included in the study if they were older than 39 years and with no evidence of cervical cytology in the previous five years within the Public Primary Health Care System in Catalonia (Spain). 1,832 underscreened women from eight public primary health areas were identified during 2007–2008 and followed-up for over three years to estimate longitudinal detection of CIN2+. Accuracy of each screening test and the combination of both to detect CIN2+ was estimated. The risk of developing CIN2+ lesions according to histology data by cytology and HPV test results at baseline was estimated using the Kaplan–Meier method.

**Results:**

At baseline, 6.7% of participants were HPV positive, 2.2% had an abnormal cytology and 1.3% had both tests positive. At the end of follow-up, 18 out of 767 (2.3%) underscreened women had a CIN2+, two of which were invasive CC. The three-year longitudinal sensitivity and specificity estimates to detect CIN2+ were 90.5% and 93.0% for HPV test and 38.2% and 97.8% for cytology. The negative predictive value was >99.0% for each test. No additional gains in validity parameters of HPV test were observed when adding cytology as co-test. The referral to colposcopy was higher for HPV but generated 53% higher detection of CIN2+ compared to cytology.

**Conclusions:**

Underscreened women had high burden of cervical disease. Primary HPV screening followed by cytology triage could be the optimal strategy to identify CIN2+ leading to longer and safe screen intervals.

## Background

Infection with high-risk human papillomavirus types (HPV) is the necessary cause for the development of cervical cancer (CC) [1]. Historically, organized screening using cytology at regular intervals with a high coverage has reduced the incidence of invasive CC in many countries [2, 3]. Absence or poor screening history remains the major risk factor for CC, and can contribute to over half of CC cases [4–9].

Primary CC screening with HPV detection has been shown in randomized controlled trials (RCTs) to have higher longitudinal sensitivity to detect cervical intraepithelial neoplasia grade 2 or worse (CIN2+) than conventional cytology, maintaining a high negative predictive value (NPV) [10–14].

In the Autonomic region of Catalonia (Spain), routine screening with cervical cytology is recommended to women aged 25–65 with a 3-year interval. Although screening is opportunistic, within the Public Health System efforts to increase CC screening coverage in underscreened women have been established [15]. These activities are facilitated by raising awareness and a campaign was launched amongst midwives, gynaecologists and family practitioners to identify poorly screened women when visiting Primary Health Care services. Women identified as being underscreened were offered a screening visit that included co-testing with HPV testing and cervical cytology in order to assure the highest accuracy of the visit. The rationale was based on the very high sensitivity and high NPV of joint testing for an extended period of three years [10–14].

The aim of this study was to evaluate the addition of HPV test to cervical cytology as a primary screening test among the underscreened population in the longitudinal prediction of CIN2+.

## Methods

**Figure 1:**
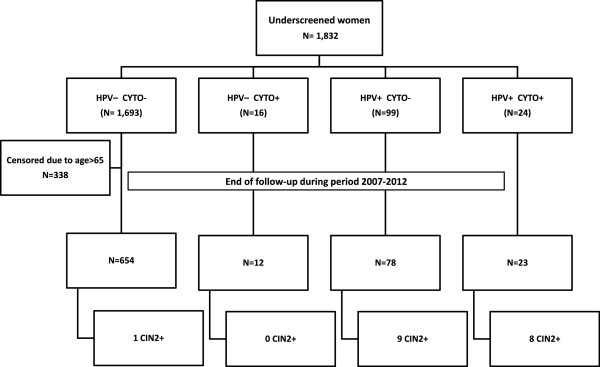
**Flowchart for the selection of the study population.** Underscreened women are defined as women older than 39 years and with no records on cervical cytology during the previous five years. CIN2+: cervical intraepithelial neoplasia grade 2 or worse.

1,832 women older than 39 years old were included. Women were selected if they had no evidence of cervical cytology in the public primary health registries in the previous five years. Women identified in eight public primary health areas of Catalonia during 2007 and 2008 were included in this study and followed-up until June 2012 (Figure 1). These women categorized as being underscreened for CC were offered cytology and HPV test at recruitment. If both tests were negative, follow-up was recommended every 3 years until age 65. Women were referred to colposcopy if either test was positive. Women older than 65 years old and with both negative tests exited the screening activity [15].

The pathology laboratories (Hospital Universitari Dr. Josep Trueta, Consorci hospitalari de Vic, Hospital Universitari Joan XXIII, Hospital del Mar, Hospital Universitari de Bellvitge, Hospital General de Granollers, Hospital d’Althaia and Laboratori d’Atenció Primària Dr. Robert) provided information on age, results and date of cytologies, histologies and HPV tests during the study period for each woman. The overall project was approved by the ethical committee of the Catalan Institute of Oncology. Any information regarding the identification of patients was anonymized before analysis.

### Screening tests

HPV detection was performed with the FDA-approved Hybrid Capture 2 test (HC2; Qiagen, Gaithersburg, MD, USA) which detects 13 high-risk HPV types (16,18,31,33,35,39,45,51,52,56,58,59 and 68). An HPV sample was considered positive if attained or exceeded the FDA-approved threshold of 1.0 pg HPV DNA ml^−1^, which corresponds to 1.0 relative light unit (RLU/CO). All HPV reference laboratories participated in an inter-laboratory quality control with kappa values over 90% [16].

Cytologies were performed largely with conventional Pap smears. Few centres used liquid based cervical cytology and in such cases the HPV and the cytology was performed in the same sample. All the cytological results were classified according to the 2001 Bethesda system [17]. Abnormal or positive cytology was defined as atypical squamous cell of undetermined significance (ASC-US) or more severe cytological diagnosis.

The CIN classification was used to categorize histological results [18].

### Follow-up

The end-point of follow-up was established at the moment of the most severe diagnosis or June 2012. For women with a positive test during the study period, a final diagnosis of “normal” was assigned if at least two negative tests were registered in subsequent visits. For women with both tests negative at baseline a subsequent negative test was requested to be categorised as being negative for CIN2+.

When concomitant cytological and histological results were available, the highest histological grade of abnormality was used for the final diagnosis. Women not having any additional test to those obtained at baseline were considered as lost to follow-up.

### Statistical analysis

Whenever appropriate, estimates are presented by different combinations of screening tests results. Three year longitudinal sensitivity, specificity, positive predictive value (PPV) and NPV for CIN2+ detection and their 95% CI were calculated for cytology, HPV test and the combination of both. Estimates were corrected taking into account the proportion of women who returned to the next screening round in each screening strata of results as reported elsewhere [19]. We estimated the risk of developing CIN2+ lesions according to histology data by cytology and HPV test result at baseline using the Kaplan–Meier method.

## Results

At baseline, the average age among the 1,832 included women was 54.1 years (range 40–88 years). Most of them (92.4%) had both tests negative. 338 women were relieved from further screening because of being 65 years old or older and had both tests negatives, leaving 1,494 undescreened women to be followed-up. Of them, 767 women (51.3%) completed follow-up. Lost to follow-up was higher in women with both tests negative when compared to those with at least one positive test (p < 0.05) (Figure 1). Increasing age was significantly associated with decreasing attendance to next screening visit (data not shown).

Positive cytology was registered in 2.2% and HPV positivity in 6.7% of the women while 1.3% had both test positive. Among HPV positive women, 19.5% had an abnormal cytology. Table 1 summarizes baseline and end of study diagnosis. At the end of follow-up nine CIN2, seven CIN3 and two invasive CC were diagnosed (18/767, 2.3%) and histologically confirmed. All but one CIN2+ were diagnosed among HPV positive women. The two CC detected corresponded to one squamous cell carcinoma (stage II) and one adenocarcinoma (stage I) and had as a baseline cytology diagnosis of ASC-US and of atypical glandular cells of undetermined significance respectively. Nine out of CIN2/3 identified during follow-up had a normal cytology at entry. The mean time between the first positive HPV test and the diagnosis of CIN2+ was 11.7 months. Among women HPV negative at enrolment 96.2% persisted as negative.

**Table 1:** **Diagnosis at follow-up among underscreened women by HPV status and concomitant cytology at baseline**

NEGATIVE HPV TEST AT BASELINE		TOTAL SAMPLE N (%)	TOTAL FOLLOW UP SAMPLE N (%)	DIAGNOSIS AT LAST FOLLOW UP						
				NORMAL N (%)	ASC-US/ASC-H N (%)	CIN1 ^a^N (%)	CIN2 ^a^	CIN3 ^a^N (%)	CERVICAL CARCINOMA ^ab^N (%)	OTHERS RESULTS ^c^N (%)
CONCOMITANT CYTOLOGY RESULT AT BASELINE	Normal	1693 (99.1)^d^	654 (98.2)	641 (98.3)^e^	1 (100)	1 (100)	1 (100)^f^			10 (90.9)
	ASC-US/ASC H/AGC/LSIL	15 (0.8)	11 (1.7)	10 (1.5)						1 (9.1)
	Suspected adenocarcinoma^g^	1 (0.1)	1 (0.2)	1 (0.2)						
	TOTAL	1709 (100)	666 (100)	652 (100)	1 (100)	1 (100)	1 (100)			11 (100)
**POSITIVE HPV TEST AT BASELINE**		**TOTAL SAMPLE N (%)**	**TOTAL FOLLOW UP SAMPLE N (%)**							
CONCOMITANT CYTOLOGY RESULT AT BASELINE	Normal	99 (80.5)^d^	78 (77.2)	45 (83.3)	6 (100)	2 (25.0)	6 (75)	3(42.9)		16 (100)
	ASC-US/AGC/LSIL	19 (15.4)	18 (17.8)	9 (16.7)		5 (62.5)	1 (12.5)	1 (14.3)	2 (100)	
	HSIL	5 (4.1)	5 (5.0)			1 (12.5)	1 (12.5)	3 (42.9)		
	TOTAL	123 (100)	101 (100)	54 (100)	6 (100)	8 (100)	8 (100)	7 (100)	2 (100)	16 (100)

^a^All the CIN1, CIN2, CIN3 and cervical cancer cases was histologically confirmed.

^b^One of the cases was an infiltrating squamous carcinoma (stage II) diagnosed at 23 months after cytology and HPV testing at baseline. The other case was an adenocarcinoma (stage I) diagnosed one month after study entry.

^c^Among negative HPV women, there were 3 cases of endometrial carcinoma who underwent a hysterectomy, 7 hysterectomies (5 for leiomyomatosis and 2 for prolapse) and one case with second positive HPV test. Among positive HPV women, there were a case with a hysterectomy for prolapse and 15 women with a persistent HPV infection.

^d^There were included in this group 23 women whose concomitant cytology at baseline had unsatisfactory results but during the follow up period, all subsequent tests were negative. There was one case in HPV positive arm.

^e^Two cases with normal concomitant and negative HPV test at baseline developed endometrial carcinoma during the follow-up period, but follow-up cytologies were normal. Another case with normal concomitant cytology and negative HPV test developed a VIN3 although Pap smears performed during the follow-up period were normal

^f^CIN2 was developed after 54 months of cytology and HPV testing at baseline. Conisation was performed but no further data was available.

^g^Finally the suspected of adenocarcinoma was a endometrial carcinoma, but follow-up cytologies were normal.

ASC-US: Atypical squamous cell of undetermined significance, ASC-H: Atypical squamous cells cannot exclude a high grade squamous intraepithelial lesion, AGC: Atypical glandular cells of undetermined significance, HPV+: positive for Human Papillomavirus test, CIN-NOS: CIN not otherwise specified, CIN1: high grade cervical intraepithelial lesions grade 1, LSIL: low grade squamous intraepithelial lesion, CIN1: high grade cervical intraepithelial lesions grade 1, CIN2: high grade cervical intraepithelial lesions grade 2, CIN3: high grade cervical intraepithelial lesions grade 3, HSIL: High grade squamous intraepithelial lesion.

**Figure 2:**
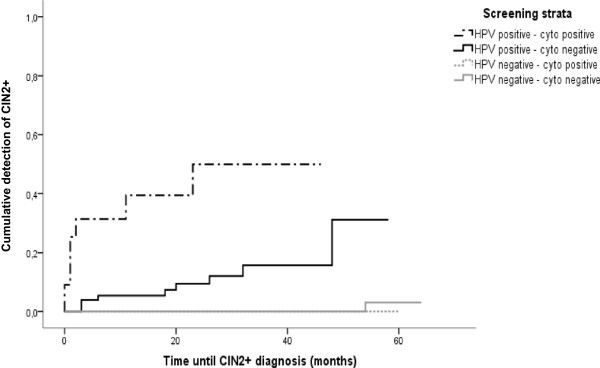
**Cumulative detection of CIN2+ according to baseline result of cytology and HPV testing.** Detection of CIN2+ in underscreened women based on 767 women. Women were classified into 4 groups depending on the HPV and cytology results at baseline. Note that there is a higher risk of development CIN2+ in positive HPV women with normal cytology.

At the end of follow-up, 27 women were classified as having non-HPV related diseases including three endometrial carcinoma cases, five leiomyomatosis and two uterine prolapses within the HPV negative strata. Among the HPV positive, 15 women had a persistent HPV infection with no further cytology data and one had a diagnosis of uterine prolapse.At 36 months, the cumulative detection of CIN2+ in women with normal cytology and HPV positive at baseline was 14.5% and 39.3% in women with both tests being positive (Figure 2).

The longitudinal sensitivity of the HPV test was considerably higher than that of cytology and equal to the combination of both tests for histologically confirmed CIN2+ (90.5; CI 95% = 88.8-92.2) (Table 2). Specificity and PPV were both higher for cytology than HPV alone or co-testing. NPV was high for both tests.

**Table 2:** **Accuracy of HPV test, cytology and the combination of both tests for CIN2+ prediction**

		HPV	CYTOLOGY	HPV + CYTOLOGY
**SENSITIVITY (95% CI)**	Uncorrected^a^	94.4 (92.8-96.1)	44.4 (39.2-49.7)	94.4 (92.8-96.1)
	Corrected^b^	90.5 (88.8-92.2)	38.2 (33.9-42.6)	90.5 (88.8-92.2)
**SPECIFICITY (95% CI)**	Uncorrected^a^	88.8 (86.4-91.2)	96.4 (95.1-97.7)	87.2 (84.6-89.7)
	Corrected^b^	93.0 (91.5-94.5)	97.8 (97.0-98.7)	91.9 (90.3-93.5)
**PPV (95% CI)**	Uncorrected^a^	16.8 (10.4-23.3)	22.9 (16.6-29.1)	15.0 (21.6-8.5)
	Corrected^b^	16.1 (11.0-21.1)	20.9 (15.9-25.8)	14.2 (9.1-19.3)
**NPV (95% CI)**	Uncorrected^a^	99.8 (99.6-100.1)	98.6 (97.8-99.5)	99.8 (99.6-100)
	Corrected^b^	99.8 (99.6-100.1)	99.1 (98.5-99.6)	99.8 (99.6-100)

^a^Estimates based only on data from women who were screened.

^b^Estimates corrected for bias due to loss of follow-up.

CIN2+: cervical intraepithelial neoplasia grade 2 or worse, hrHPV: HPV testing for high-risk types, PPV: positive predicted value, NPV: negative predicted value, 95% CI: 95% Confidence Interval.

HPV detection was performed with Hybrid Capture 2 test (HC2; Qiagen, Gaithersburg, MD, USA).

## Discussion

CC screening activities aim to reach asymptomatic women in specific target ages on regular basis. The organization of these activities and the quality control measures are crucial to optimize the resources for the best health benefits. Irrespective of the type of the screening offered, poor attendance by some and over use of the system by others are persistent issues that need to be addressed. Our principal aim was to identify among poorly screened women in a single screening visit any possible cervical intraepithelial lesion that could lead to cancer. To provide the best optimal detection at the time of the visit, HPV was offered as an ancillary test to the cytology to increase longitudinal sensitivity with a minimal impact in specificity. This intervention differed from that given to the regular users of screening facilities in which only cytology was offered every three years or two consecutive cytologies within one year if it was the first screening visit [15].

Co-testing with HPV referred 7.6% of underscreened women for an either closer follow-up or to immediate colposcopy. If we had used previous guidelines, in which cytology was the screening test, all the women with a first normal cytology at baseline (97.8%) had to be screened again within a year in order to correct for poor cytology sensitivity. This was not implemented in our population because of the co-testing recommendation.

Positive cytology was detected in 2.2% of the women, percentage that was overall similar to that observed in the general population of the same age group (2.1%), although lesions in our study were more severe than expected [20]. However, by using HPV test, 6.7% of the women were positive but we identified over 50% more CIN2+ in the three years following the index screening visit than with the solely use of cytology. The difference in cumulative risk of CIN2+ for those with a double negative tests results compared to those with a HPV-negative test was minimal (0.2%) supporting the fact that, in this population, single testing with HPV could be sufficient as the first screening test. These results were consistent with the state of the art knowledge provided by several RCTs comparing clinically validated HPV tests with cytology as primary screening tests [10–12, 14, 21]. Further, meta-analysis of studies using HC2 as HPV test, reached an overall longitudinal sensitivity of 96.3% and a specificity of 91.4% for CIN2+ detection, the latter being slightly lower than the one observed for cytology [13]. In our data we observed a loss of 4.8% in longitudinal specificity when using HPV test alone compared to cytology alone. To avoid this drop in specificity appropriate algorithms must be implemented as triage tests such as reflex cytology or HPV genotyping for HPV16 or 18 and others [12, 13, 22, 23].

The cumulative detection of CIN2+ among women with normal cytology at baseline was high for HPV positive women at baseline compared to those HPV negative and comparable to that seen in other screening cohorts [12, 24]. Our findings suggest that the main benefit of HPV testing is the identification of women harbouring clinically relevant lesions [12, 25, 26]. In fact, studies with longer follow-up periods confirm that HPV positive women with a normal cytology harbour an increased risk in the long run of CIN2, CIN3, and invasive CC and that an increased over-detection of HPV tests can be ruled out [27]. An increased number of referral tests due to an excess of positive HPV tests in women with no disease could be an undesired effect of this strategy [26, 28]. In three European RCTs about seven women had a potential false-positive screening result for each CIN2+ detected [26]. In our study, this ratio was 6.3 women for each CIN2+ or 14 women for each CIN3+ detected. In the ATHENA HPV trial, a screening strategy with HPV testing followed by a reflex cytology, resulted in 4.5 colposcopies per CIN2+ detected, similar to the rate of using HPV with genotyping [29]. Total number of colposcopies for CIN2+ detected in screened women is now considered a good quality indicator of overdiagnosis [30]. However, in the POBASCAM trial, the number of referrals in the HPV positive arm was considerably reduced in further screening rounds if the interval was long enough to avoid detection of acute HPV infection [25]. Efforts to minimize referrals should not only be an economical aim but also a good clinical practice aim to avoid unwanted effects of screening such as overtreatment or anxiety associated to a positive test.

European RCTs and American screening cohorts have shown that among HPV negative women, the risk for CIN2+ was very low (0.2% and 1.2%, for women without or with cytological abnormalities respectively) suggesting that safe intervals can go beyond five years if a validated HPV tests is being used [13, 25, 26, 31, 32] providing a beneficial cost-efficacy ratio [33]. In our study, 97.8% of the HPV negative women, irrespective of the cytology result, the risk for CIN2 was 0.2% and 0% for CIN3+, reassuring a safe 3-year screening interval.

In this study we have explored the strategy to protect poorly screened women by introducing a more complete screening approach. However, screen negative women were prone to a poor follow-up at 3 years as almost half of them did not return during the follow-up period consistently with that observed in other studies [14]. A behavioural study in the region identified that the large majority of poorly screened women reported poor knowledge about the relevance of CC screening [34] indicating that efforts to explain the benefits of screening should be reinforced.

Due to the differential attendance to control visits according to the screening baseline results we corrected the accuracy parameters (i.e. sensitivity, specificity) by follow-up estimates to minimize any bias [19]. We could not correct for a potential verification bias as HPV-negative women were not referred to colposcopy and biopsy. But in our study, about 7% of women with negative screening results had histological data for unknown reasons to the investigators. Unfortunately we did not have any more details on other medical reason of why these women were biopsied. However, none of these women were diagnosed with CIN2+. In Kulasingam et al. study, in which random biopsies were performed in all double negative women, no CIN2+ was reported [19]. Thus, the data support that if there is any identification bias the weight of it must be small.

We were concerned about the low longitudinal sensitivity of the cytology test but it is well accepted that quality of cytology depends on many factors as is extremely amenable to poor reproducibility. Although a relevant proportion of the women were menopausal, we did not find differences by age strata or by pathology laboratory but our sample size was relatively small and estimates by strata were unstable.

Strengths of this study are the performance of screening and follow-up processes in many centres across Catalonia as part of the routine CC screening. Co-testing with cytology and HPV allowed us not only the comparison between tests but also to speculate about different screening scenarios as testing was done blind to the other test result. Finally, the HPV testing used complied with its recognized clinical validity and reproducibility [16].

## Conclusions

In a group of underscreened women participating in opportunistic screening, HPV test, as primary screening tool, was superior to cytology for CIN2+ detection with a higher longitudinal sensitivity over 3-year follow-up. Both tests had a very high NPV. Primary HPV screening followed by cytological triage could be the optimal strategy to identify CIN2+ in poor screening attenders in developed countries leading to longer and safe screen intervals.

## Abbreviations

CC: Cervical cancer
CIN2+: Cervical intraepithelial lesion grade 2 or worse
HPV: HPV testing for high risk HPV types
RCTs: Randomized Controlled Trials
95% CI: 95% confidence intervals
NPV: Negative predicted value
PPV: Positive predicted value.
